# Intensified summer monsoon and the urbanization of Indus Civilization in northwest India

**DOI:** 10.1038/s41598-018-22504-5

**Published:** 2018-03-09

**Authors:** Yama Dixit, David A. Hodell, Alena Giesche, Sampat K. Tandon, Fernando Gázquez, Hari S. Saini, Luke C. Skinner, Syed A. I. Mujtaba, Vikas Pawar, Ravindra N. Singh, Cameron A. Petrie

**Affiliations:** 1 Godwin Laboratory for Palaeoclimate Research, Department of Earth Sciences, University of Cambridge, Cambridge, CB2 3EQ United Kingdom; 2 IFREMER, Unité de Recherche Géosciences Marines, Z.I. Pointe du Diable, BP 70, 29280 Plouzané, France; 3 Department of Earth and Environmental Sciences, IISER Bhopal, India; 4 School of Earth and Environmental Sciences, University of St. Andrews, St. Andrews, UK; 5 Geological Survey of India, Faridabad, India; 6 Department of History, Maharshi Dayanand University, Rohtak, Haryana India; 7 Department of AIHC and Archaeology, Banaras Hindu University, Varanasi, India; 8 Department of Archaeology, University of Cambridge, Cambridge, CB2 3DZ United Kingdom; 9 Present Address: Earth Observatory of Singapore, Nanyang Technological University, 50 Nanyang Avenue, 639798 Singapore

## Abstract

Today the desert margins of northwest India are dry and unable to support large populations, but were densely occupied by the populations of the Indus Civilization during the middle to late Holocene. The hydroclimatic conditions under which Indus urbanization took place, which was marked by a period of expanded settlement into the Thar Desert margins, remains poorly understood. We measured the isotopic values (δ^18^O and δD) of gypsum hydration water in paleolake Karsandi sediments in northern Rajasthan to infer past changes in lake hydrology, which is sensitive to changing amounts of precipitation and evaporation. Our record reveals that relatively wet conditions prevailed at the northern edge of Rajasthan from ~5.1 ± 0.2 ka BP, during the beginning of the agricultural-based Early Harappan phase of the Indus Civilization. Monsoon rainfall intensified further between 5.0 and 4.4 ka BP, during the period when Indus urban centres developed in the western Thar Desert margin and on the plains of Haryana to its north. Drier conditions set in sometime after 4.4 ka BP, and by ~3.9 ka BP an eastward shift of populations had occurred. Our findings provide evidence that climate change was associated with both the expansion and contraction of Indus urbanism along the desert margin in northwest India.

## Introduction

**Figure 1:**
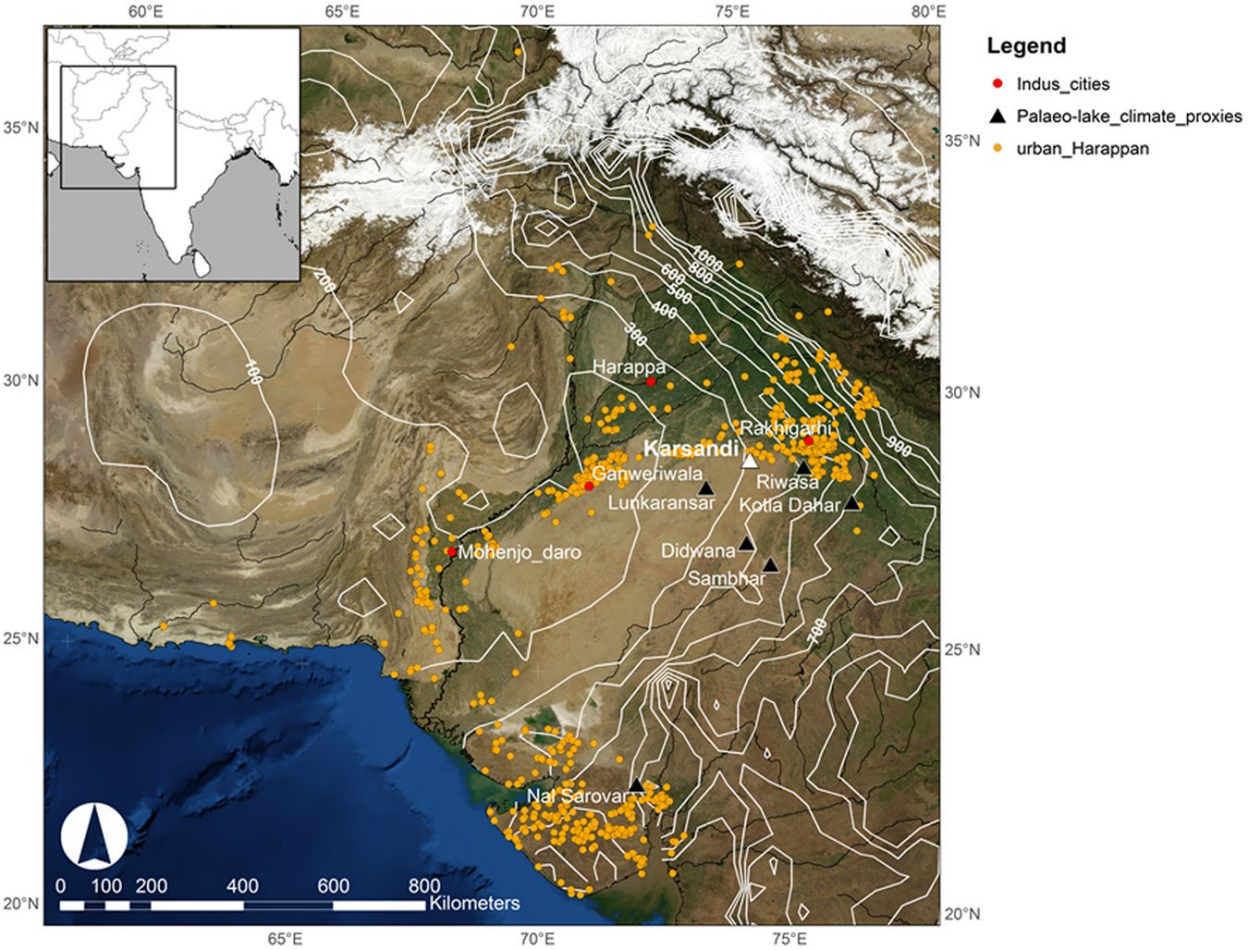
Map of NW India showing the location of paleolake Karsandi (white triangle) and other paleolake records (black triangles). Orange circles denote the Indus settlements in northwest India and the red dots are the urban Indus centres. White lines are isohyets (mm) between 1900 and 2008. Inset shows location of the main map in relation to the limits of the Indian subcontinent. Rainfall isohyets were extracted from the University of Delaware monthly global gridded high resolution station (land) data set of precipitation from 1900–2008 (v2.01). Data available for free from: http://www.esrl.noaa.gov/psd/data/gridded/data.UDel_AirT_Precip.html UDel_AirT_Precip data provided by the NOAA/OAR/ESRL PSD, Boulder, Colorado, USA, from their Web site at https://www.esrl.noaa.gov/psd/. NASA Blue Marble: Next Generation satellite imagery data freely available at NASA's Earth Observatory (NASA Goddard Space Flight Center http://earthobservatory.nasa.gov/Features/BlueMarble/). Maps composed using Esri ArcGIS 10.2.0.3348.

Paleolake Karsandi is located in the arid Nohar-Bhadra district in the Indian state of Rajasthan on the Thar Desert margin in northwest India (Fig. 1). At present, this region is a desert erg characterized by aeolian landforms, including sand dunes and ridges with intervening sand sheets, with sparse depressions and deflation hollows^1^. Today the area is devoid of drainage or active lakes; however, paleolake deposits provide evidence for wetter climate conditions at times during the Holocene^1^. There is also evidence for intermittent human occupation along the margin of the Thar Desert in Cholistan, Pakistan, and northern Rajasthan and Haryana in northwest India during different periods^2–6^. Populations of the Indus Civilization occupied these areas from about five thousand years before present (ka BP) and they went on to create urban centres around ∼4.6–4.5 ka BP^7^. Following a peak in occupation, urban decline began from ~4.1 to 4.0 ka BP^7,8^, and it has been suggested that this process may have been affected by the weakening of the Indian Summer Monsoon (ISM)^9–15^. The areas occupied by the Indus populations were, however, characterized by climatic and ecological diversity^16^. Consequently, it is important to reconstruct the local climate in the areas occupied to fully understand human adaptation, sustainability and resilience to a changing climate^16^. There is a range of previous studies from lake deposits in the Thar Desert^17–19^, but they present an inconsistent history of climate variability because the lakes are located in different precipitation zones within the Thar Desert and there are difficulties with establishing accurate chronologies^20^. Here we present the mid-Holocene hydroclimate history from the edge of the northern Thar Desert using the isotopic composition (δ^18^O and δD) of gypsum hydration water (GHW)^21^ deposited in paleolake Karsandi in northwest India (Fig. 1).

The paleolakes of the Thar Desert that have been previously investigated lie in different climatic zones, stretching from the arid zone that receives 100–250 mm/year rainfall in the west to regions of increasing rainfall including the semi-arid (250–500 mm) and semi-humid (500–600 mm) zones towards the east^19,20^ (Fig. 1). The rainfall in this broad region is derived primarily from the Indian summer monsoon. Paleolake Karsandi lies in the semi-arid zone on the NE Thar Desert margin. It is in the vicinity of several important Indus settlements including Rakhigarhi (which lies ~120 km to the northeast), and the smaller centres of Kalibangan (~100 km northwest) and Karanpura (~40 km northeast). The Karsandi region currently receives annual rainfall of ~300 mm, ~80% of which falls under the influence of the Bay of Bengal arm^22^ of the south-westerly summer monsoon from June to September^23,24^. The remaining rain falls in winter from November through March, when N-NW winds bring relatively dry air to the region. The mean monthly air temperatures range from 17 to 32.9 °C with a minimum average monthly temperature of 5 °C during January and a maximum of 45 °C during May (recorded in Hissar for the period 1983–2012, 120 km east of Karsandi). Evapotranspiration averages ~2000 mm/year with a maximum during May and June^1^, resulting in a strongly negative hydrologic balance.

**Figure 2:**
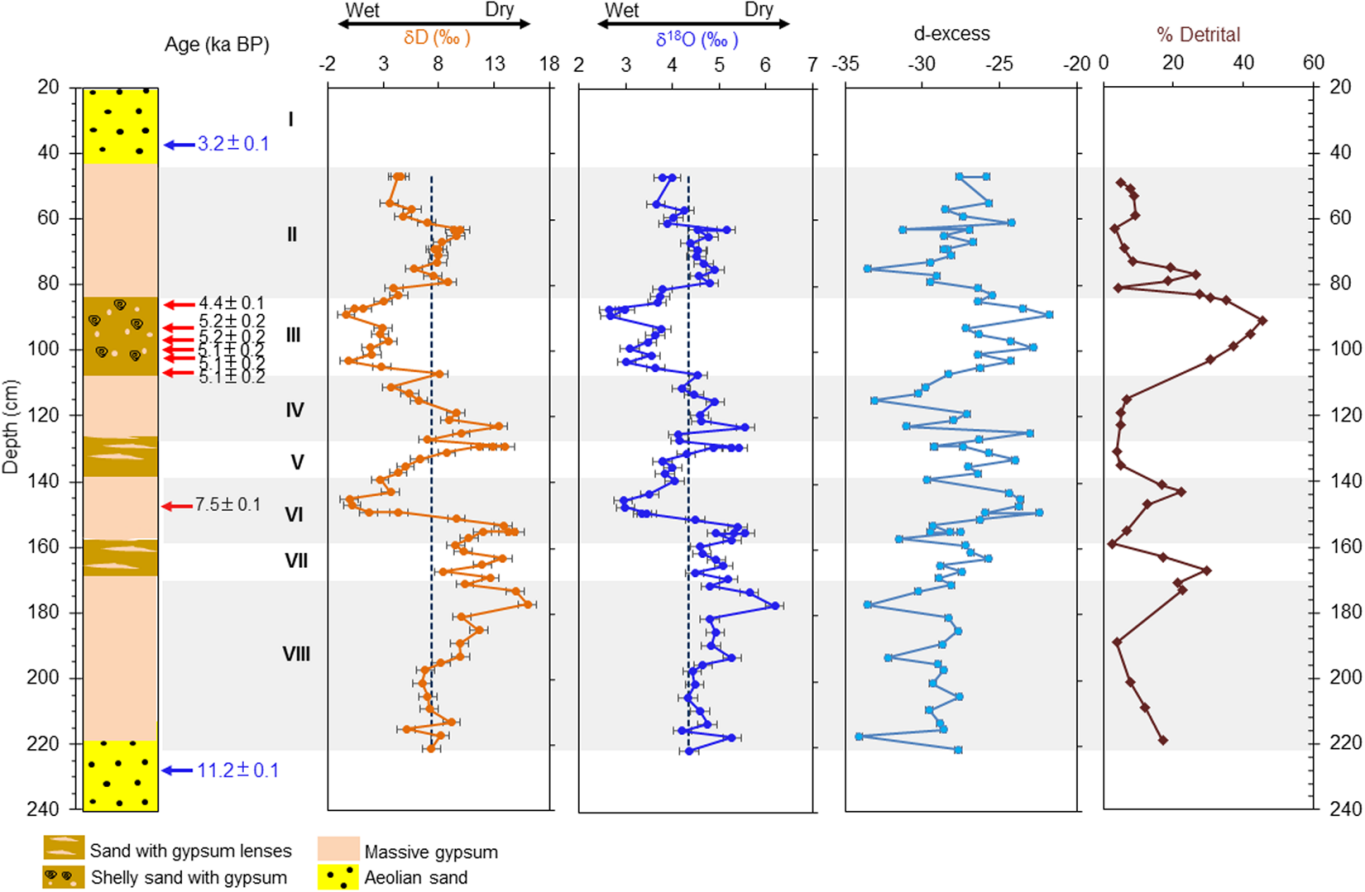
Lithostratigraphy of the sediment section and δD, δ^18^O, d-excess of paleolake Karsandi water obtained after correction of GHW isotopes for fractionation factors. Detrital content (% detrital) is shown in brown. Black vertical line denotes the mean isotopes across the section. The calibrated radiocarbon ages (ka BP) are shown in black with red arrows pointing to their respective depths. OSL dates and depth of sand collection for dating are shown in blue. Grey bands denote the nearly pure gypsum deposits indicating periods of relatively lower rainfall. Roman numerals denote lithologic units.

The lithostratigraphy of paleolake Karsandi was described initially by Saini *et al*.^1^. We sampled an exposed, 2.2-m sediment section near Karsandi village (N28°59′29.6: E74°45′53.8) (Fig. S1). The section comprises six units of alternating massive gypsum and gypsiferous sand deposited between two aeolian sand units that have been dated at ~11.2 and 3.2 ka BP by OSL (Fig. S1). The gypsum deposits at Karsandi are matrix free, well-sorted gypsum crystals exhibiting primary features – i.e., clear, euhedral crystals, growing in clusters. We suggest that the gypsum is primary and has retained its isotopic composition because of ensuing arid conditions^19^ and the fact that the deposits were not deeply buried. We measured the stable isotopes of gypsum hydration water (GHW) in samples taken every 2 cm along the sediment section and the chronology was determined using radiocarbon and OSL dates (see Methods for details) (Figs 2, S2 and Table 1).

**Table 1:** Karsandi OSL and radiocarbon analyses. Radiocarbon dates were calibrated using CALIB software using the IntCal13 data set^42^.

Depth (cm)	OSL Lab ID	Material	Age cal yr BP	Error (years)
35–40	KS-2	Aeolian sands	3200	100
230	KS-1	Aeolian sands	11200	100
**Depth (cm)**	**Lab Sample ID**	**Material**	**Radiocarbon age** ^**14**^**C yrs B.P**.	**Calibrated age (2σ) yrs B.P**.
88–91	G1482/4	Terrestrial gastropods	3969 ± 35	4297–4525
91–93	G1478/1	Gastropods fragments	4561 ± 39	5050–5440
93–97	G1479/3	Gastropods fragments	4541 ± 49	5040–5440
99–103	6125.1.1	Gastropod fragments	4362 ± 71	4828–5261
103–105	G1480/2	Terrestrial gastropods	4401 ± 40	4860–5270
105–107	G1481/5	Terrestrial gastropods	4445 ± 44	4870–5290
150	156771	Chara	6600 ± 30	7433–7565

## Results and Discussion

Karsandi was a closed playa lake with inflow primarily through precipitation either directly onto the playa basin or via surface runoff or subsurface flows from rainfall elsewhere in the watershed. Hydrological loss was dominantly by evaporation. Sediments consists of aeolian sands at the top and bottom of the section with alternating massive gypsum units interbedded with sand units consisting of detrital (quartz) and gypsum grains. Owing to the relatively low solubility of gypsum in water (~2.5 g/l)^25^, gypsum precipitated from the lake water when climate conditions were dry and the hydrologic budget had higher rates of evaporation over precipitation (E/P). Massive gypsum precipitated when the playa lake was maintained at gypsum saturation throughout the year, through low inflow (both surface and groundwater) and high evaporation rates (Fig. S3). In addition to autochthonous gypsum, Karsandi paleolake’s sediments also contain allochthonous detrital grains (mainly quartz) transported by winds or surface water inflow to the lake during periods of greater rainfall (Fig. S4).

**Figure 3:**
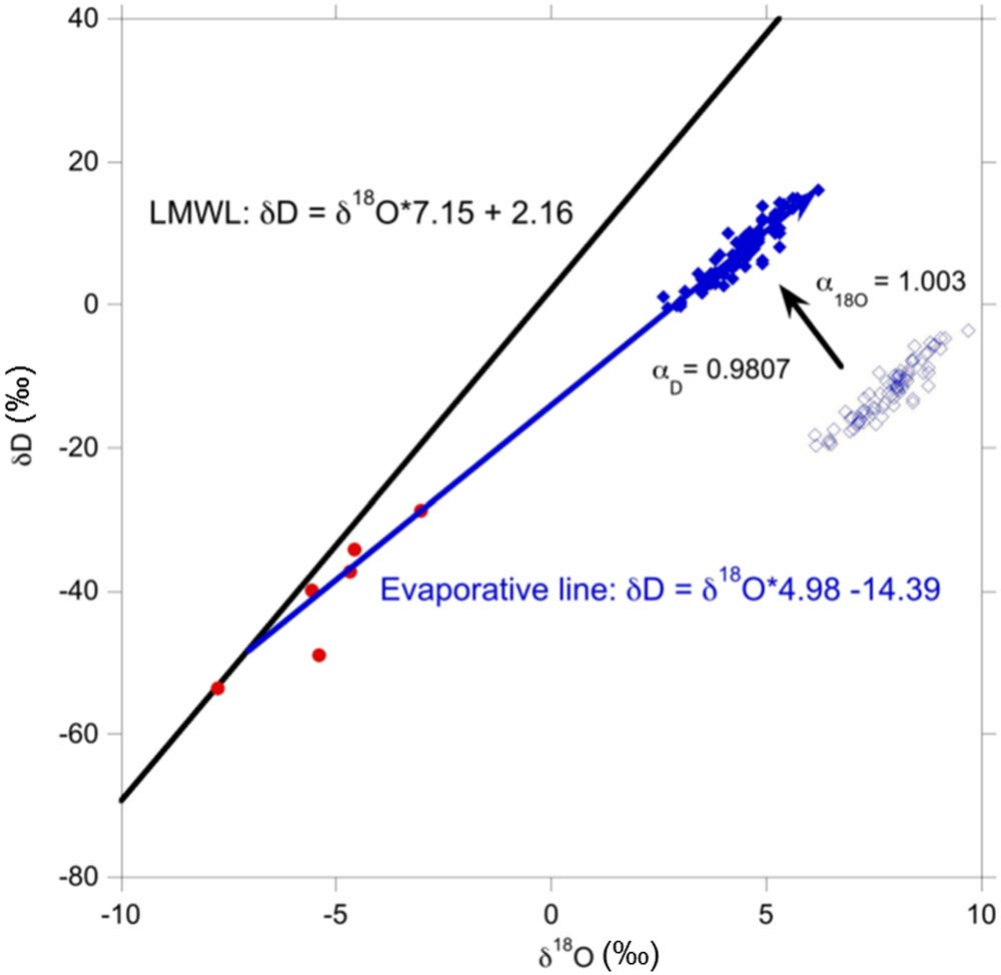
δ^18^O and δD of gypsum hydration water as measured from Karsandi paleolake (open blue diamonds), and predicted paleo-lake water values for Karsandi (closed blue diamonds) after correction for fractionation factors^27^. The local meteoric water line (LMWL) is based on GNIP data from Delhi^32^. The solid blue line represents the evaporative line estimated from the corrected Karsandi data. Surface water samples (red filled circles) from nearby Riwasa village^28,29^ are plotted for comparison.

We infer changes in E/P using both the sediment composition and isotopic composition (δ^18^O and δD) of hydration water of lacustrine gypsum deposits^21,26^. When corrected by known fractionation factors^27^, the measured δ^18^O and δD of GHW directly reflect the isotope composition of the paleolake water from which the gypsum formed, provided no recrystallization or isotopic exchange has occurred following deposition. The calculated paleolake water values plot on an evaporative line of slope 5^28^, which is consistent with a few surface water samples collected from Riwasa village (situated ~170 km east) (Fig. 3). The Karsandi paleolake water line intercepts the local meteoric water line at δ^18^O and δD equal to −7.1‰ and −48.7‰, respectively, which is reasonable for regional groundwater.

The δ^18^O and δD of Karsandi lake water depends on the isotopic composition of the precipitation (input), which is related to the monsoon intensity, and the amount of water lost to evaporation (E/P in the region)^29^. We interpret lower isotopic (δ^18^O and δD) values of GHW as periods of increased monsoon rainfall and lower E/P, whereas periods of decreased monsoon precipitation and higher E/P are marked by relatively high δ^18^O and δD values of GHW.

The d-excess (δD − 8*δ^18^O) is a derived parameter that mainly reflects the temperature and relative humidity (RH) conditions under which evaporation of lake water occurred. Greater d-excess values generally imply evaporation at higher effective RH and vice-versa.

Near the base of the section, prior to ~11.2 ± 0.1 ka BP, arid conditions prevailed, as indicated by the presence of aeolian sands. After ~11.2 ± 0.1 ka BP, sediments (Unit VIII) consist of massive gypsum (~95% gypsum), indicating the appearance of a shallow evaporative lake associated with the early Holocene strengthening of the Indian summer monsoon^29^ that was forced by increased boreal summer insolation related to Earth’s precessional cycle^29,30^. The appearance of this shallow lake at Karsandi coincided with the filling of nearby paleolake Riwasa at ~11.1 ka BP^28,29^. It is also consistent with pollen profiles and geochemical results from other Rajasthani lakes including Lunkaransar, Didwana, Bap-Malar and Kanod, which record the development of water bodies in the Thar Desert following the last glacial period^18,31^ (Figs S5, S6). From the Early Holocene until ~5.1 ± 0.2 ka BP (Units VIII-IV), relatively drier climate prevailed at Karsandi as compared to the mid-Holocene, with intermittent wetter periods marked by increased delivery of detrital sediment and lower water isotope values.

During the mid-Holocene, beginning at ~5.1 ± 0.2 ka BP (Unit III), the detrital content of the sediments began to increase and the gypsum content decreased, suggesting increased clastic deposition as rainfall increased (Fig. 2). This phase of deposition is associated with a sharp decline in δ^18^O and δD values of GHW, indicating increased monsoonal rainfall into the lake catchment (Fig. 2). The d-excess in Unit III increases with decreasing δ^18^O and δD values of GHW. Since the d-excess of input precipitation from New Delhi (the nearest GNIP station) decreases during the summer monsoon when δ^18^O and δD decrease^32^, we therefore conclude that the d-excess during this period is controlled by the conditions of evaporation over Lake Karsandi and not the d-excess of the input (rainfall). The increasing d-excess with RH in Unit III also supports evaporation under more humid conditions than subsequent Unit II.

Unit III was also characterized by the presence of terrestrial gastropods that lived in the littoral zone suggesting intervals of desiccation during the dry season. A species of freshwater ostracod belonging to the genus *Cyprinotus* was also found, suggesting development of a freshwater lake during periods of greater rainfall. Apart from *Cyprinotus* sp., no other ostracod species were found. *Cyprinotus* is known to have durable eggs that can withstand desiccation, but hatching of eggs only occurs under aquatic conditions, thereby permitting survival during times of unfavorable conditions^33^. The presence of freshwater ostracod-bearing sands and gypsum with low δ^18^O and δD values together indicates that this period was characterized by an environment where greater monsoon rainfall results in groundwater and surface flow into the lake in summer season transporting detrital sediments followed by gypsum precipitation during the dry season (Fig. S3). The lower δ^18^O and δD in gypsum hydration water during the deposition of Unit III can be attributed to the strengthened summer monsoon rainfall, which was quite depleted in heavier isotopes because of the ‘amount effect’ and consequently gave the lake water a lower δ^18^O and δD signature. During the dry season, evaporation started with this low initial δ^18^O and δD composition of the lake water and the playa lake reached gypsum saturation state. The isotopic composition of playa lake water was, however, still quite low after evolution following the Rayleigh fractionation, which led to gypsum precipitation with lower hydration water isotopes in Unit III (see supplementary information for details). This is in contrast to the periods of massive gypsum deposition, when the summer monsoon was weaker and the rainwater had relatively heavier initial isotopic composition to start with. The low inflow throughout the year (winter and summer) and evaporation maintained the playa lake at gypsum saturation and continuous gypsum with high δ^18^O and δD precipitated.

**Figure 4:**
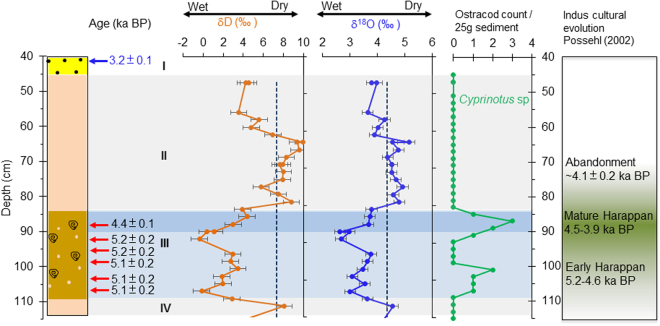
Correlation of climatic variability recorded in the lithostratigraphy, δD (orange), δ^18^O (blue), of paleolake Karsandi water and ostracod abundance with Indus cultural changes. The calibrated radiocarbon ages (ka BP) are shown in black with red arrows pointing to their respective depths. OSL dates and depth of sand collection for dating are shown in blue. Grey bands denote the nearly pure gypsum deposits indicating periods of relatively lower rainfall and blue band denotes wetter periods. Roman numerals denote lithologic units. The Early phase of the Indus Civilization developed during increased monsoon intensity as indicated by lower GHW isotopes and high shell abundance after ~5.1 ± 0.2 ka BP. The Mature Harappan phase and peak in urbanism coincides with the lowest GHW isotopes and highest shell abundance between ~5.0 and ~4.4 ka BP. Note that the subsequent decline in urbanism and disappearance of Post-urban Harappan sites in this region is coincident with drying conditions suggested by reappearance of massive gypsum with increasing GHW isotopes and complete absence of ostracod and gastropod shells.

The timing of increased monsoon rainfall from ~5.1 ± 0.2 ka BP appears to precede the expansion of Early Harappan populations in northwest India, which included the occupation of the Thar Desert fringe^3,4,6^ (Fig. 4). Notably, the results from Karsandi suggest that the climate after ~5.1 ± 0.2 ka BP was wetter than present on the Thar Desert margin, which potentially made the region more habitable for these Indus populations. The evidence for increased monsoon rain in the Karsandi catchment from ~5.1 ± 0.2 ka BP broadly coincides with evidence for increased settlement in the surrounding region and the Cholistan Desert margin from ~5.0 ka BP. The δ^18^O and δD of GHW from Karsandi further decreases to lowest values in Unit III averaging ~3 and 1.6 ‰, respectively between 5.0 and 4.4 ka BP (Fig. 4), suggesting further strengthening of monsoon rainfall and decreased E/P in the Karsandi catchment. This period was also marked by maximum abundance of the ostracod *Cyprinotus* sp. Although there is uncertainty in both the archaeological and paleoclimate chronologies, the wettest period at Karsandi (Unit III) overlaps with the village-based Early Harappan phase and the rise of the Indus urban centres from ~4.6–4.5 ka BP^7,34^. Our results thus indicate that the northern Thar Desert margin experienced increased precipitation at the time of the development of the Indus cities such as at Rakhigarhi, located ~120 km northeast from Karsandi.

Lacustrine sequences from the Thar Desert have been used previously to infer the paleoenvironmental history of the region. For example, Enzel *et al*.^17^ studied lake Lunkaransar sediment chemistry and concluded that relatively dry conditions prevailed during the period of Indus urbanism, challenging the so-called ‘culture-climate’ hypothesis^35^, which purports that Indus urbanism developed under wetter climatic conditions. Lake Lunkaransar is located ~120 km southwest of Karsandi in the arid zone (200–250 mm/year rainfall) of the Thar Desert and its lake level declined at ~5.3 ka BP^17^. Similarly, other lakes from the arid western Thar Desert, Lake Bap Malar and Kanod dried out from ~5.5 ka BP. These dates for the development of arid conditions are significantly earlier than those from lakes Karsandi and Didwana, which lies to its east^31^ and maintained high stands until ~4.4 ka BP^18^. In contrast, Lake Sambhar situated at the easternmost margin of the Thar Desert had high lake levels until ~2.5 ka BP^36^ (Fig. S6). The drying of lakes in the Thar Desert thus exhibit a diachronous pattern of east-west drying following the modern precipitation gradient^37^ (Fig. 1). The location of paleolake Karsandi on the northeastern fringe of the Thar Desert and its proximity to clusters of Indus archaeological settlements makes it an important archive for inferring local climate history.

The transformation of sandy Unit III to the massive gypsum of Unit II indicates the reappearance of a shallow saline lake. The δ^18^O and δD of GHW progressively increase to an average of 4.2‰ and 5‰, respectively, and ostracods and gastropods are absent from this gypsum unit. The exact timing of the beginning of gypsum Unit II cannot be determined owing to lack of datable material, but it occurred sometime after 4.4 ± 0.1 ka BP and continued until ~3.0 ka BP when the topmost aeolian sand was deposited (Fig. 4, Table 1). The timing of the onset of drying conditions is, however, coincident or perhaps slightly earlier than evidence for a weakening of the monsoon at 4.1 ± 0.1 ka BP observed at Kotla Dahar^8^. Furthermore, the span of this drier period is concurrent with the decline and ultimate abandonment of the Indus urban centres, and much of the subsequent post-urban phase. Previous paleoclimate studies using lacustrine sediments from Haryana^10^, marine sediments from the Arabian Sea^12^, and a speleothem from northeast India^9^ all document a decline in summer monsoon rainfall at about 4.1 ka BP, which has been linked to Indus de-urbanization^16^. Although admittedly the age control is poor in Unit II, a Bayesian age model^38^ with the available OSL and radiocarbon ages (Figs. S5,S6) suggest the timing of the high δ^18^O and δD values of GHW in Unit II at Karsandi is estimated to be at ~4.1 ± 0.1 ka BP, which indicate that this region experienced drier conditions at this time. By ~3.2 ± 0.1 ka BP, the Karsandi playa lake dried up permanently and aeolian sand deposition continued at this location until the present day, suggesting the monsoon weakened to its modern level in this region.

**Figure 5:**
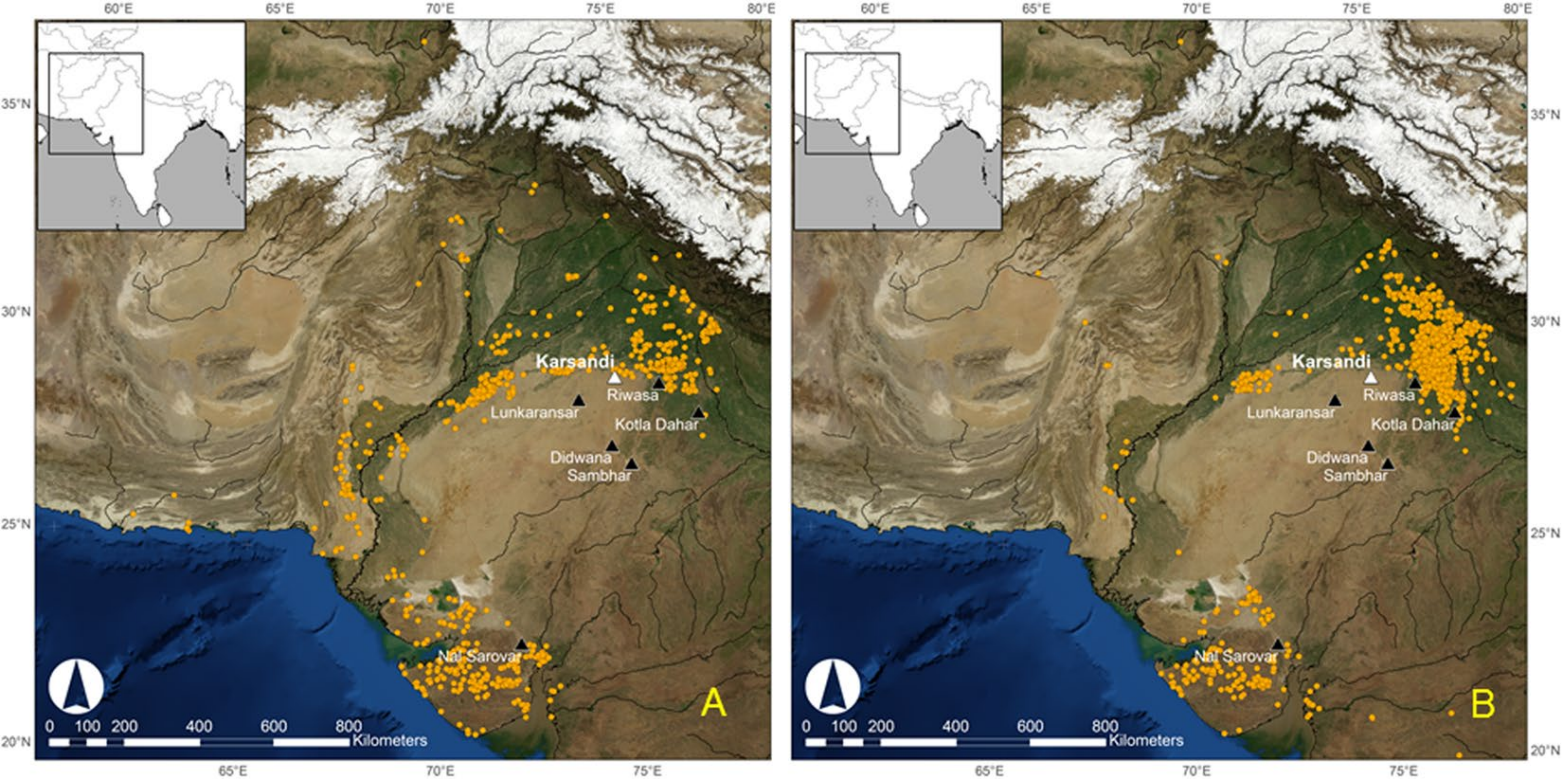
Location of (**A**) Urban Harappan sites at ∼4.6–4.5 ka BP and (**B**) Post- Urban Harappan after ~4.1–4.0 ka BP sites in NW India as denoted by the orange dots in each case. Note that the urban-Harappan sites are located on the margin of the Thar Desert and the post-urban Harappan sites are clustered to the right of paleolake Karsandi on the Indo-Gangetic plains. The location of Karsandi shown by the white triangle and other reported paleolakes in black triangles. Rainfall isohyets were extracted from the University of Delaware monthly global gridded high-resolution station (land) data set of precipitation from 1900–2008 (v2.01). Data available for free from: http://www.esrl.noaa.gov/psd/data/gridded/data.UDel_AirT_Precip.html. UDel_AirT_Precip data provided by the NOAA/OAR/ESRL PSD, Boulder, Colorado, USA, from their Web site at https://www.esrl.noaa.gov/psd/. NASA Blue Marble: Next Generation satellite imagery data freely available at NASA's Earth Observatory (NASA Goddard Space Flight Center http://earthobservatory.nasa.gov/Features/BlueMarble/). Maps composed using Esri ArcGIS 10.2.0.3348.

Current archaeological evidence suggests that the process of Indus deurbanization after ~4.1–4.0 ka BP included a reduction in settlement density in the western and central parts of the zone occupied by Indus populations and an increase in the number of village–sized settlements in its eastern reaches^16^ of NW India (Fig. 5). Although the exact age of the onset of aridity could not be ascertained, the paleolake Karsandi record supports drier climatic conditions on the NE Thar Desert margin sometime after ~4.4 ± 0.1 ka BP. Madella and Fuller^16^ have previously attributed the shifts in settlements in the Late Harappan transition partly to agricultural readjustments potentially resulting from changes in climatic conditions^39^. However, cultural transformation is a complex process and changing climatic conditions in the region may have been one of several factors that affected cultural behavior and the available subsistence choices made by Indus populations^13^.

## Conclusions

It is increasingly evident that the landscapes across which Indus populations lived were diverse in terms of climate, geology and ecology, and the patterns of cultural behavior and response to climate variability are unlikely to have been uniform throughout the Indus region^16,24^. The paleoclimate record from paleolake Karsandi clearly suggests there were areas receiving favorable rainfall in the period leading up to the development of Indus urban centres along the northern fringe of the Thar Desert in NW India. This evidence underscores the importance of reconstructing local conditions for understanding the degree of adaptation and resilience of ancient civilization exhibited to climate change.

## Methods

### Gypsum hydration water

Gypsum crystals were picked for a total of 71 samples that ranged from depths 47–221 cm in the sequence. For each sample, 150–200 mg of gypsum was ground into a fine powder using an agate mortar, and then dried overnight at 45 °C. GHW was recovered by heating the powdered gypsum *in vacuo* using a bespoke offline extraction system consisting of six vacuum lines contained within a modified gas chromatography (GC) oven in the Godwin Laboratory at the University of Cambridge (UK), using the method described in Gázquez *et al*.^26^. Weight loss of the gypsum was measured to ensure the purity of the gypsum and verify that all water (theoretically 20.9% of the total weight of gypsum) was extracted during the procedure. An average weight loss of 20.0 ± 0.7% was recorded for all gypsum samples. Oxygen (δ^18^O) and hydrogen (δD) isotopes of the hydration water were measured simultaneously by CRDS using a L2140-i Picarro water isotope analyzer and A0211 high-precision vaporizer at the Godwin Laboratory at the University of Cambridge^26^. The same CRDS instrument is equipped with a Micro-combustion module (MCM; Picarro inc.) that was filled with a pyrolytic catalyst for the removal of organics in the water as the organic compounds in GHW can spectroscopically interfere with the CRDS analyses^26^. Internal standards were calibrated against V-SMOW, GISP, and SLAP for δ^18^O and δD. All results are reported in parts per thousand (‰) relative to V-SMOW. The external error of the method was ±0.2‰ for δ^18^O and ±0.8‰ for δD, as estimated by repeated analysis (n = 26) of an internal gypsum standard extracted together with the samples in the hydration water extraction apparatus.

### Chronology

The sediment section was dated by radiocarbon measurements of aragonitic gastropod shells and calcitic charophyte gyrogonites by Accelerator Mass Spectrometry (AMS) (Table 1). Typically, ~2–3 complete gastropods and 15–20 charophyte gyrogonites were used for AMS dating. Radiocarbon samples were measured at the Queens University, Belfast and Lawrence Livermore National Laboratory, Berkeley. Gastropod shell fragments from horizons from 91–93 cm, 93 to 97 cm and 99 to 103 cm from Unit III were merged because the material was insufficient for radiocarbon analysis. Prior to target preparation at CAMS, shells were gently leached in dilute hydrochloric acid (1N) to remove the surface layer that is susceptible to diagenetic alteration. We used terrestrial gastropods for radiocarbon dating and assume the reservoir effect was minimal because the pulmonate (lung breathing) land snails record atmospheric CO_2_. Because of the absence of carbonate outcrops in the region and the fact that bedrock is composed mainly of granitic and rhyolitic rocks^40,41^, the contribution of dead carbon from the catchment is assumed to be small. In addition, the large surface area to volume of this shallow lake system should promote CO_2_ equilibration with the atmosphere (Broecker and Walton, 1959). Radiocarbon dates were calibrated using CALIB program the IntCal13 data set^42^ (Table 1). Calibrated ages are expressed as kiloyears before present (ka BP) over a 2σ-error range. Additionally, aeolian sands from the top and bottom of the Karsandi lacustrine deposits were dated using Optically stimulated luminescence (OSL) dating at the Geological Survey of India, Faridabad (Table 1, see supplementary information for detailed methodology).

## Electronic supplementary material

Supplementary Information
