# Author Correction: Intensified summer monsoon and the urbanization of Indus Civilization in northwest India

**DOI:** 10.1038/s41598-018-30084-7

**Published:** 2018-08-03

**Authors:** Yama Dixit, David A. Hodell, Alena Giesche, Sampat K. Tandon, Fernando Gázquez, Hari S. Saini, Luke C. Skinner, Syed A. I. Mujtaba, Vikas Pawar, Ravindra N. Singh, Cameron A. Petrie

**Affiliations:** 1 Godwin Laboratory for Palaeoclimate Research, Department of Earth Sciences, University of Cambridge, Cambridge, CB2 3EQ United Kingdom; 2 IFREMER, Unité de Recherche Géosciences Marines, Z.I. Pointe du Diable, BP 70, 29280 Plouzané, France; 3 Department of Earth and Environmental Sciences, IISER Bhopal, India; 4 School of Earth and Environmental Sciences, University of St. Andrews, St. Andrews, UK; 5 Geological Survey of India, Faridabad, India; 6 Department of History, Maharshi Dayanand University, Rohtak, Haryana India; 7 Department of AIHC and Archaeology, Banaras Hindu University, Varanasi, India; 8 Department of Archaeology, University of Cambridge, Cambridge, CB2 3DZ United Kingdom; 9 Present Address: Earth Observatory of Singapore, Nanyang Technological University, 50 Nanyang Avenue, 639798 Singapore

Correction to: *Scientific Reports* 10.1038/s41598-018-22504-5, published online 09 March 2018

In Figure 4 the Ostracod curve does not align with δD (orange) and δ^18^O (blue) curves. The correct Figure 4 appears below as Figure 1.

**Figure 1:**
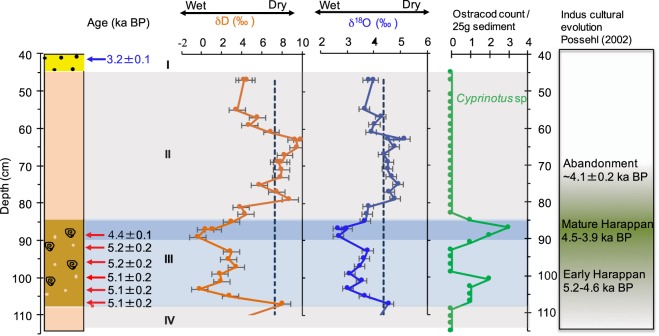
Correlation of climatic variability recorded in the lithostratigraphy, δD (orange), δ^18^O (blue), of paleolake Karsandi water and ostracod abundance with Indus cultural changes. The calibrated radiocarbon ages (ka BP) are shown in black with red arrows pointing to their respective depths. OSL dates and depth of sand collection for dating are shown in blue. Grey bands denote the nearly pure gypsum deposits indicating periods of relatively lower rainfall and blue band denotes wetter periods. Roman numerals denote lithologic units. The Early phase of the Indus Civilization developed during increased monsoon intensity as indicated by lower GHW isotopes and high shell abundance after ~5.1 ± 0.2 ka BP. The Mature Harappan phase and peak in urbanism coincides with the lowest GHW isotopes and highest shell abundance between ~5.0 and ~4.4 ka BP. Note that the subsequent decline in urbanism and disappearance of Post-urban Harappan sites in this region is coincident with drying conditions suggested by reappearance of massive gypsum with increasing GHW isotopes and complete absence of ostracod and gastropod shells.

