# Supplementary material for: Intensified summer monsoon and the urbanization of Indus Civilization in northwest India
Source: Sci Rep. 2018 Mar 9;8:4225. doi: 10.1038/s41598-018-22504-5 (PMC5844871; doi:10.1038/s41598-018-22504-5)
Supplement: Supplementary file 1 — Supplementary Information [file 41598_2018_22504_MOESM1_ESM.docx]

**Intensified summer monsoon and the urbanization of Indus Civilization in northwest India**

Yama Dixit^1,2^^[[1]](#footnote-1)^*, David A. Hodell^1^, Alena Giesche^1^, Sampat K. Tandon^4^, Fernando Gázquez^1,3^, Hari S. Saini^5^, Luke Skinner^1^, Syed A.I. Mujtaba^5^, Vikas Pawar^6^, Ravindra N. Singh^7^, Cameron A. Petrie^8^

^1^Godwin Laboratory for Palaeoclimate Research, Department of Earth Sciences, University of Cambridge, Cambridge, CB2 3EQ, United Kingdom

^2^IFREMER, Unité de Recherche Géosciences Marines, Z.I. Pointe du Diable, BP 70, 29280 Plouzané, France

^3^School of Earth and Environmental Sciences, University of St. Andrews, UK

^4^Department of Earth and Environmental Sciences, IISER Bhopal, India

^5^Geological Survey of India, Faridabad, India

^6^ Department of History, Maharshi Dayanand University, Rohtak, Haryana, India

^7^ Department of AIHC and Archaeology, Banaras Hindu University, Varanasi, India

^8^Department of Archaeology, University of Cambridge, Cambridge, CB2 3DZ, United Kingdom

Supplementary information

OSL dating methodology

The middle part from each sample tube was selected to determine the depositional ages by OSL technique ^1^. The outer parts were used for dose rate and water content measurements. A selected portion was treated with 1N HCl solution to remove the carbonates and 30% H2O2 to remove organic impurities. The dried sediments were sieved to separate a 90-150 μm grain size fraction from which quartz grains were separated using a sodium polytungstate solution of 2.58 g/cm^3^ specific density. The 20 μm outer layer of quartz grains was removed by etching with 40% HF for 80 minutes and then by HCL for 20 minutes. This was done to remove feldspars and the alpha irradiated layer. The quartz grains were temporarily mounted on stainless steel discs (9.6 mm diameter) as a monolayer, generally over a circular area of 4 mm diameter with the help of silicon spray. Luminescence measurements were carried out using a Risoe TL/OSL Reader 15-20 DA, equipped with a calibrated Sr-90 beta source. The stimulation was made using blue LED’s (470±30 nm) and the luminescence was detected in the UV range through a Hoya UV-340 filter placed in front of an EMI9671 photomultiplier tube. Luminescence was recorded in 250 channels (40 s) in which counts of first five channels were considered for further calculation after subtracting background counts of the last 25 channels. The aliquot discs were placed in alternating holes of the sample carousel to avoid irradiation/stimulation crosstalk. Generally four pre-dating tests were conducted on each sample before actual measurement of De. These included feldspar screening, rough dose estimation, preheat plateau test and dose recovery test. When feldspar signals were recorded, the sample was retreated with HF. Generally the IRSL/OSL ratio was kept below 1%. Nevertheless, an additional step of IRSL at 75°C per sec for 100 sec was given before each blue OSL measurement. Blue light stimulation was carried out at 125°C for 40 s. A preheat temperature of 220°C for 10 s and TL of 180°C (in-lieu of preheat) were found suitable. The heating rate used was 5°C/s. The dose recovery was within ±10% of the administered dose. The equivalent dose (De) was measured following the Single Aliquot Regeneration (SAR) protocol ^2,3^.Sample discs with recycling ratio within 10% and recuperation of less than 5% were considered for final calculation of the De values. The commercial software “Analyst” was used for the calculation of individual De values. Calculation of De’s was repeated from the spreadsheets provided by A.S. Murray, Risoe National Laboratory, Denmark. It was found that average De values of well bleached samples were similar in both calculations. The spreadsheets were helpful in discriminating the recuperation, sensitivity changes and recycling ratio. The samples had near normal distribution of De values, therefore, weighted averages were employed for age calculation using Grun software. The dose rates (Dr) of samples were calculated from the concentration of U and Th (analyzed by ICP-MS) and K (by flame photometer at Chemical Labs, GSI, Faridabad). The OSL ages are compiled in Table 1.

**Isotope mass balance model for Unit III gypsum deposition**

As described in the main text, paleolake Karsandi was a closed basin during the Holocene and the only significant water loss from the lake was through the process of evaporation. The change in oxygen isotope ratios from a drying water body can be described by the Craig-Gordon equation^4^, which takes into account evaporation and the changing isotopic composition between lake and atmospheric water in similar lines as Dixit et al., 2016^5^. The assumption made in the calculations is that the water loss results from evaporation and that the conditions of evaporation (e.g., temperature, relative humidity) remained unchanged. The evolution of oxygen isotope ratios as a function of fraction of residual lake water with progressive evaporation is calculated from the following relationship ^6^:

where δ_0_ is the initial isotopic composition of water, *f* is the fraction of residual water in the lake, A/B is the isotopic composition that water attains in its final evaporation stages when *f* approaches zero and A and B are:

δ_a_ is the δ^18^O of the atmosphere, which can be calculated from the ambient temperature in Karsandi as, δ^18^O = 0.39T (°C) – 22.8;

Given the average annual temperature range at Karsandi, 17 to 33°C, the calculations were performed at 25°C, a_w_ is the thermodynamic activity of water: a_w_ = -0.000543/*f^2^* -0.018521/*f* + 0.99931; *h* is the fractional relative humidity, taken as h/a_w_ that changes with lake volume; ∆ε, the kinetic enrichment factor: ∆ε= 0.0142 (1-h/a_w_); α is the equilibrium fractionation factor: α = exp (1137/*T^2^* - 0.4156/*T*- 0.00207) ^7^; T is the temperature in Kelvin; ε is total isotopic enrichment factor: ε= α-1

We evaluate two possible scenarios firstly for Unit III deposition (Case I) and secondly for the deposition of massive gypsum (Case II).

**Case I: When the summer monsoon rain was higher than today (in blue).** We take the initial composition of lake water, δ_0_ = -10, the present day isotopic composition of the summer rain.

**Case II: When the summer monsoon rainfall was much lower (in red)** and therefore initial composition of lake water δ_0_ = -7, which is about the present day isotopic composition of the groundwater in Karsandi (obtained from the intersection of Karsandi evaporative line and the local meteoric water line as described in the main text).

Using these two initial composition of lakewater as the starting point, the calculations for the evolution of δ^18^O in the lake water were carried out until *f =* 0.2. The δ^18^O in the lake water obtained by evaporation of lake water until the remaining volume changed from 100 to 20%, is ~3‰, which is about the average δ^18^O of Unit III and is lower than the δ^18^O of gypsum units.


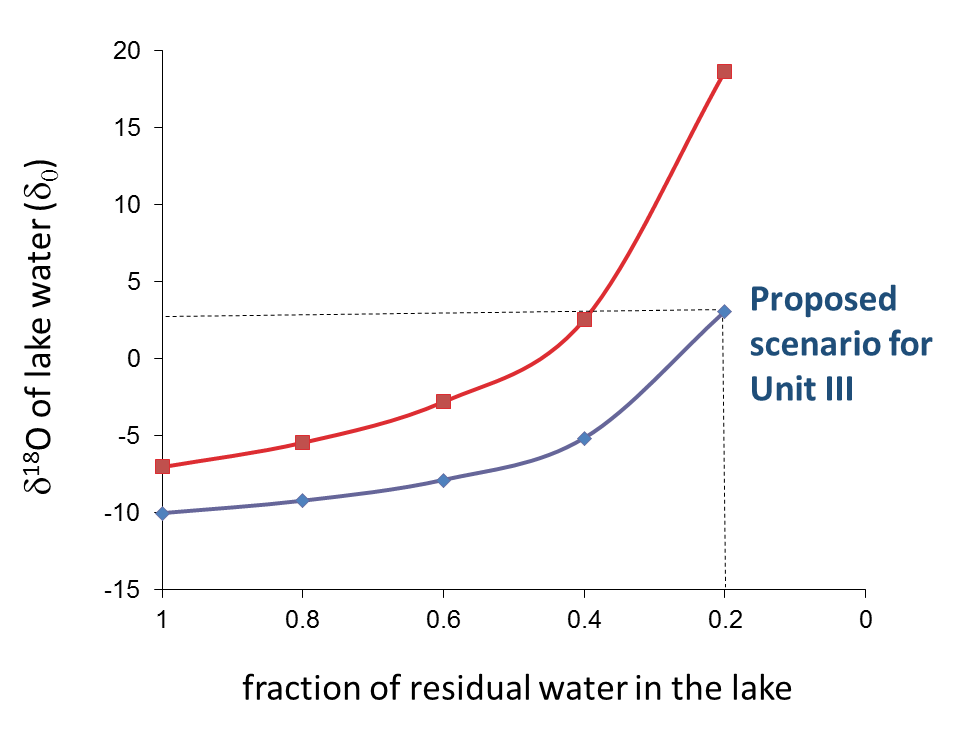


Supplementary Figure 1


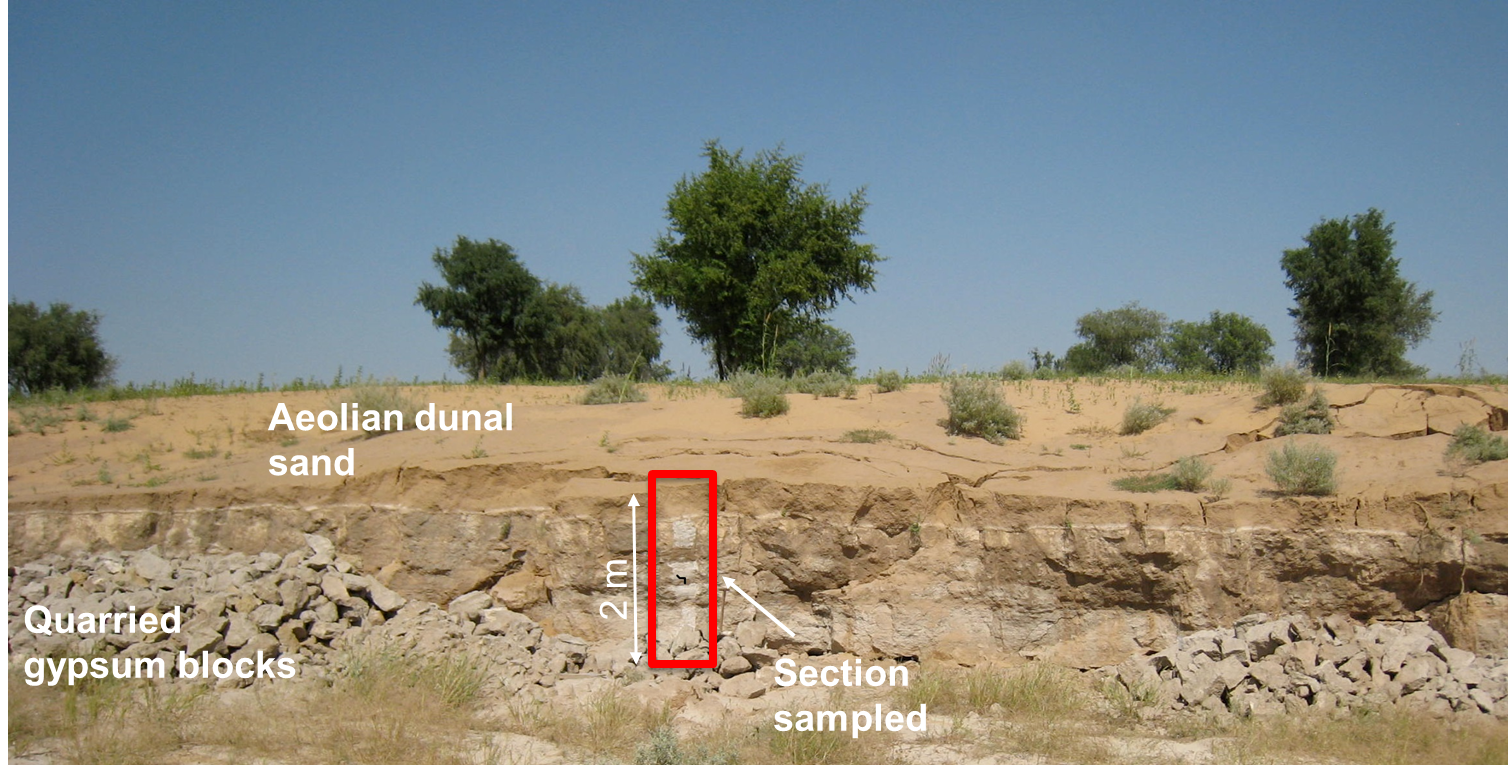


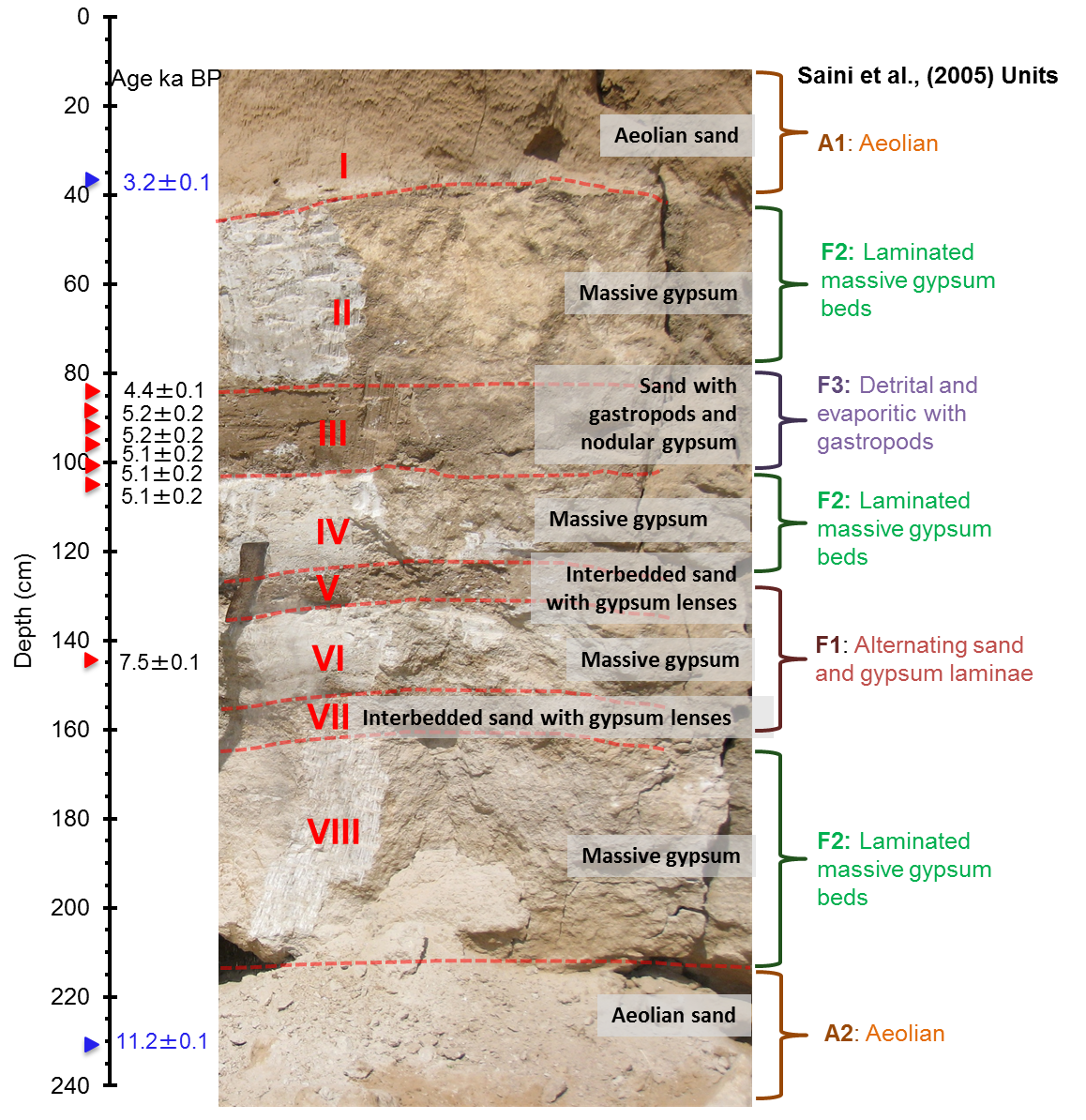


**Figure S1.** The top image shows a panoramic view of the location of paleolake Karsandi deposits (N28°59’29.6: E74°45’53.8) from Nohar-Bhadra district in the Indian state of Rajasthan on the Thar Desert margin in northwest India. The red rectangle denotes the approximate size of the section sampled for this study. Bottom figure shows the lithology of Karsandi paleolake section sampled (this study) and corresponding units (A1, A2, F1-F3) as described by Saini et al (2005)^8^. Position of radiocarbon levels are denoted by red triangles and OSL dates in blue. Unit I- Yellow- brown Aeolian sand; Unit II- Massive gypsum deposits; Unit III- silty sand with nodular gypsum and ostracods and gastropods, Unit IV- Massive gypsum; Unit V- Interbedded sand with gypsum; Unit VI- Massive gypsum; Unit VII Interbedded sand with gypsum lenses; Unit VIII- Massive gypsum deposits. The lacustrine deposits from Unit I-VIII are underlain by yellow brown aeolian sand.**Supplementary Figure 2:**


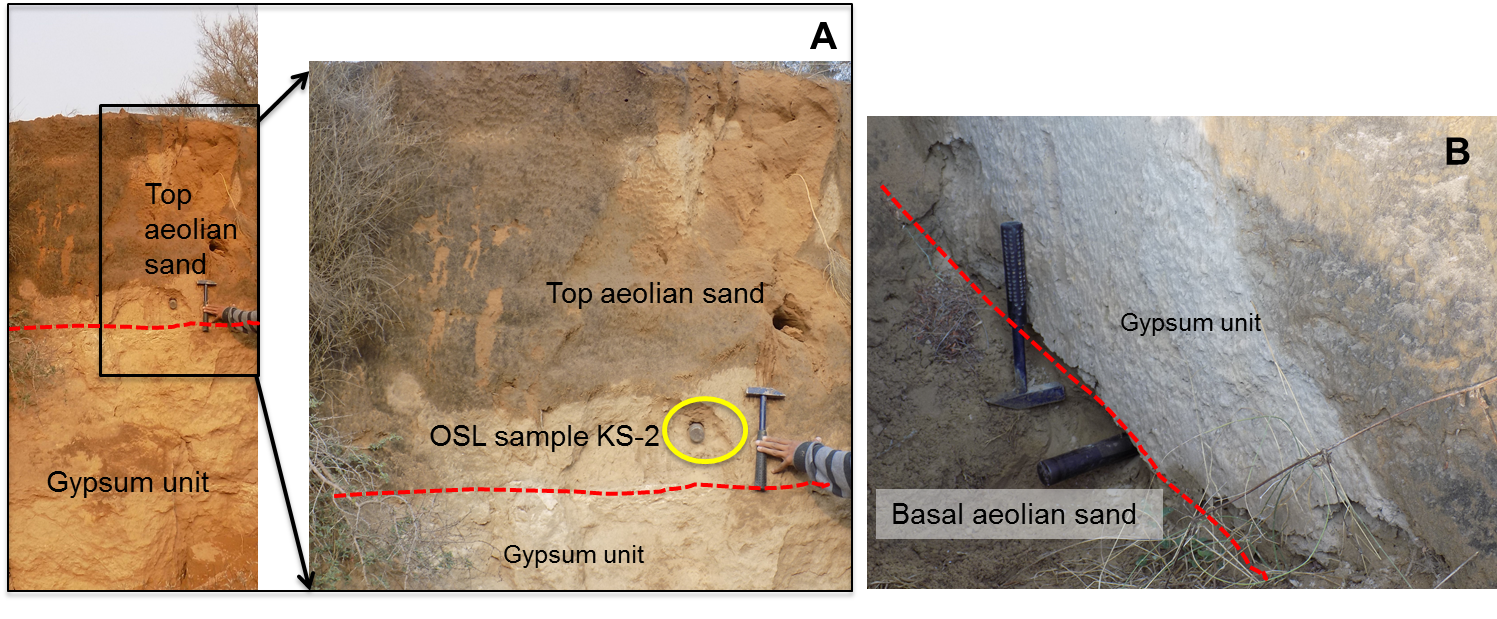


**Figure S2:** (A) Location of samples taken for OSL dating located just above the topmost gypsum unit at 40 cm; (B) Location of OSL date sample taken from the aeolian sands underlying the lowermost gypsum unit at 230 cm.

**Supplementary Figure 3**

**
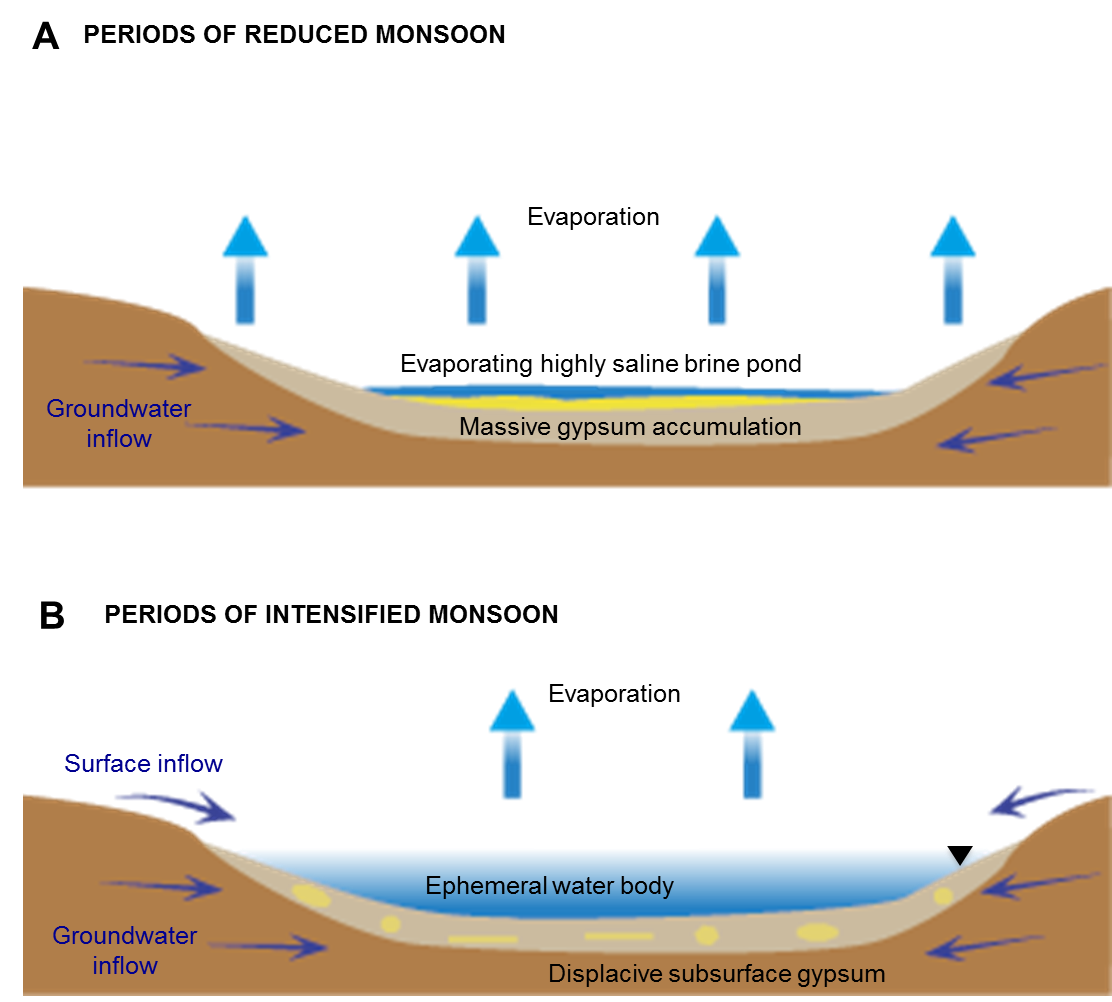
**

**Figure S3.** Schematic cross-section of an ephemeral playa (using CorelDRAW X6 software). (A) During periods of reduced monsoon, a shallow saline pond is maintained at gypsum saturation throughout the year through groundwater inflow to the playa basin and high evaporation rates, resulting in massive continuous gypsum deposits. (B) In contrast, periods of greater monsoon rainfall results in groundwater and surface flow into the lake in summer transporting detrital sediments followed by gypsum formation during the dry season.

# Supplementary Figure 4

**
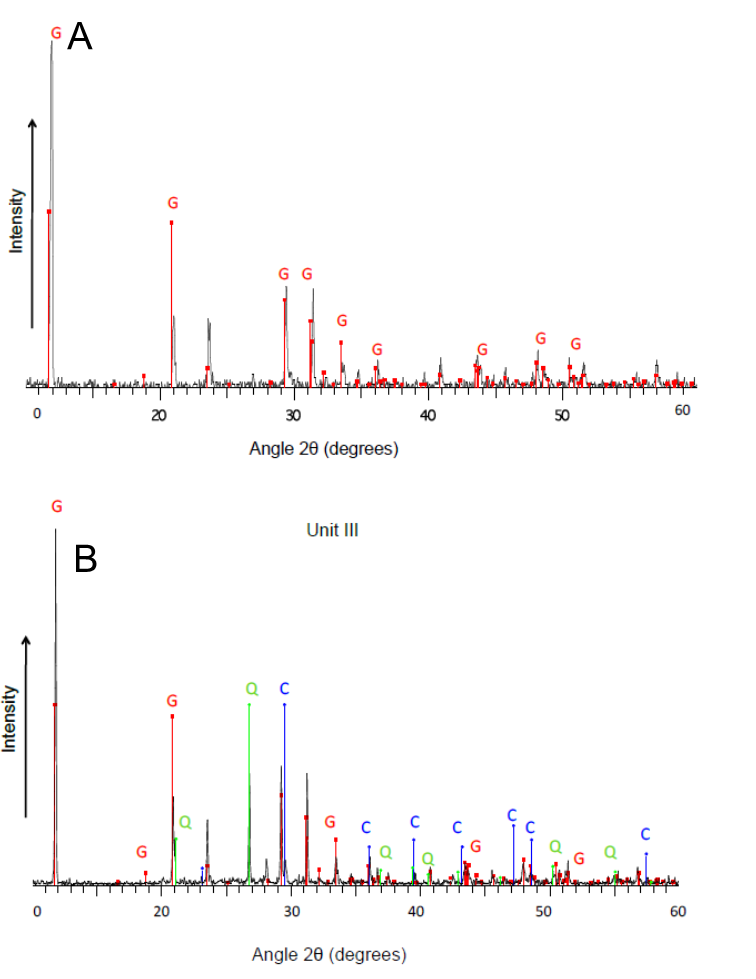
**

**Figure S4.** XRD results of (A) bulk sediment sample from a typical gypsum unit from Karsandi, Unit II (10-12 cm) showing 2-theta peak of gypsum. (B) bulk sediment sample from Unit III (12-14 cm) showing 2-theta peak of gypsum (G), calcite (C) and quartz (Q). The reference peak positions and intensities are from the PDF2 database. Mineral identification was aided by the use of an automated search-match computer program^9^.

# Supplementary Figure S5

**
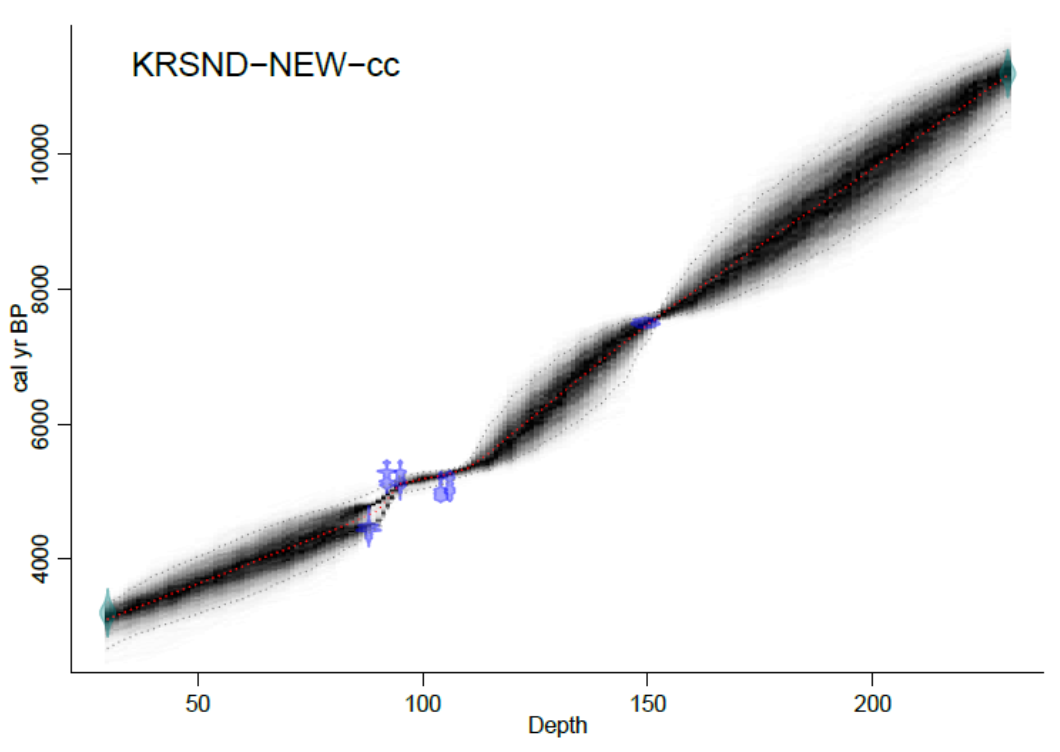
**

**Figure S5.** Karsandi age model obtained using BACON program based on flexible Bayesian age modeling ^10^. The top and bottom OSL dates are shown in light blue colour. The radiocarbon dates were calibrated using the IntCal13 calibration curve ^11^.

**
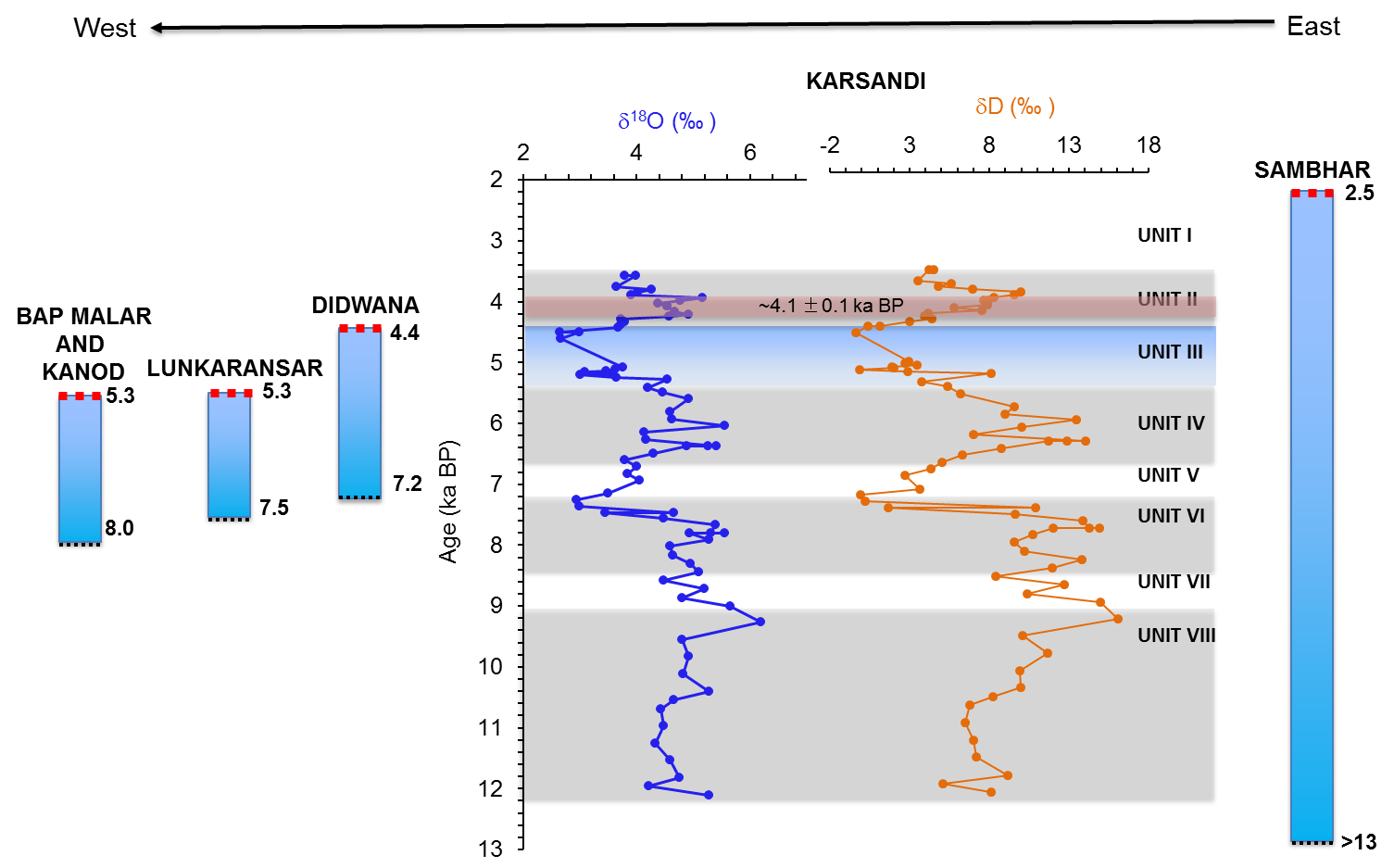
**

**Figure S6.** δ^18^O and δD values of lake water plotted vs BACON model ages. Grey bands show the gypsum units. The wettest period suggested by high δ^18^O and δD values occurs between ~5.2 and ~4.4 ka BP. High δ^18^O and δD values indicating arid conditions (shown in red bar) has a median age of ~4.1 ± 0.1 ka BP, which is coincident with the timing of Indus de-urbanization ^12^. Also shown are the high lake level period (rectangles in blue) and timing of permanent decline of lake levels (indicated by red dashed line) in other Thar Desert lakes from west to east, Lakes Bap Malar ^13^ and Lunkaransar ^14^ on the western margin dried up first followed by Didwana ^15^, Karsandi and finally Sambhar ^16^. The numerals indicate ages in ka BP.

# Supplementary Figure S7

#
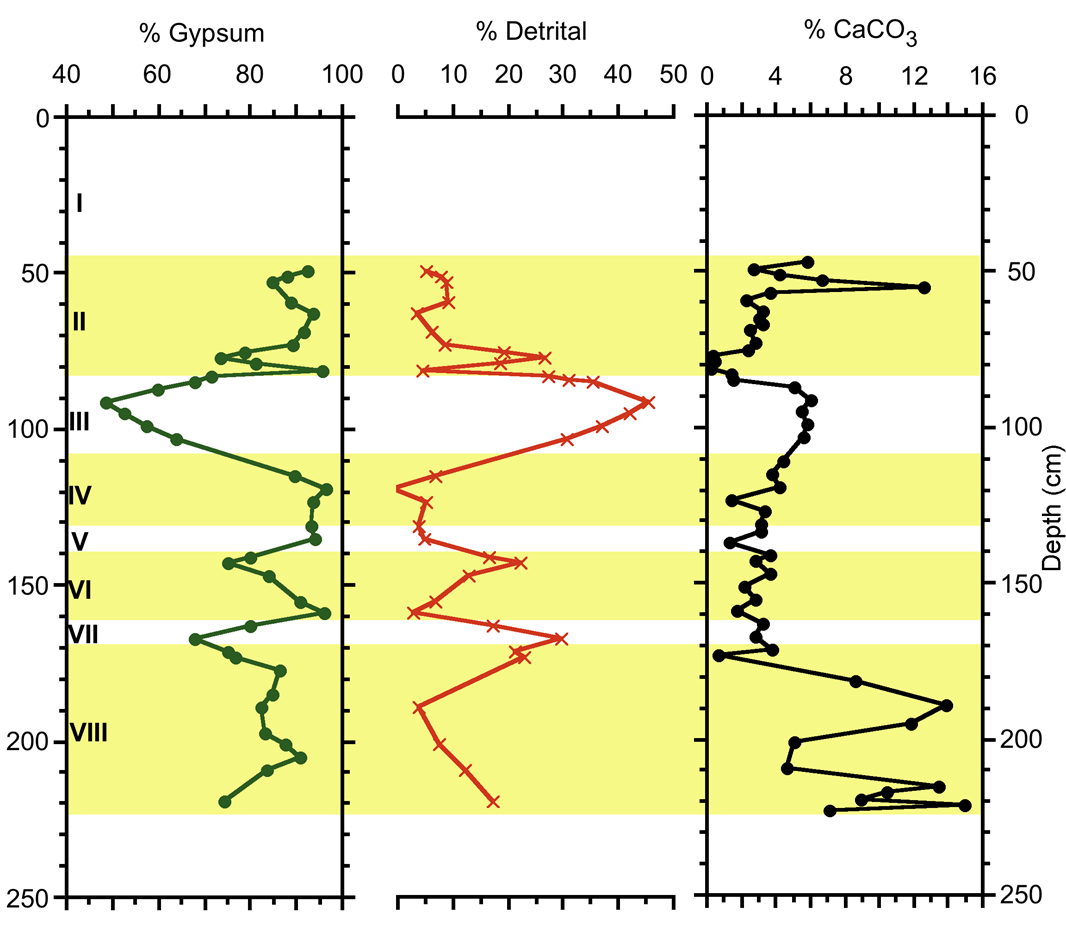


**Figure S7:** Percent gypsum (green), % detrital (red) and % calcium carbonate (black) from paleolake Karsandi. Yellow horizontal bars denote the gypsum units VIII, VI, IV and II.

**Supplementary Figure S8**


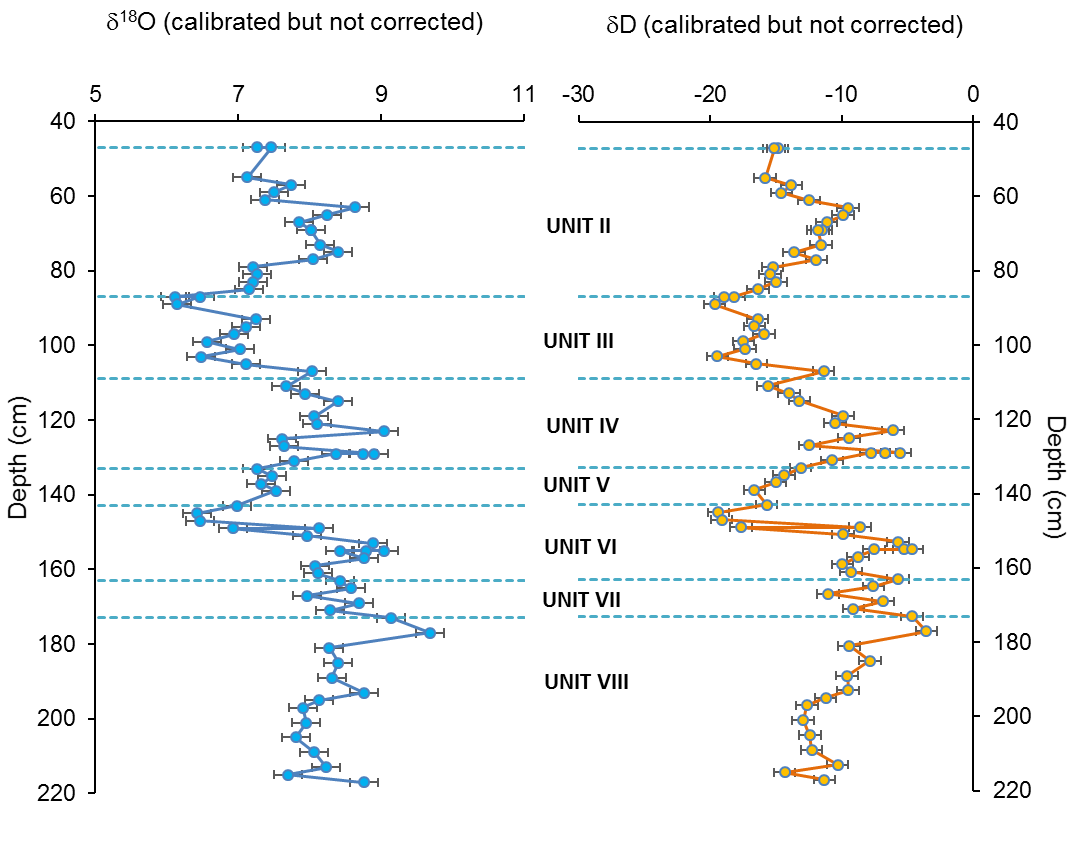


**Figure S8**: Uncorrected δ^18^O and δD of gypsum hydration water from Karsandi profile. Error bars show ± 1σ based on the δ^18^O and δD results for the internal gypsum standard NewGyp ^17^.

**References:**

1. Aitken, M. J. *Introduction to optical dating: the dating of Quaternary sediments by the use of photon-stimulated luminescence*. (Clarendon Press, 1998).

2. Murray, A. S. & Roberts, R. G. Measurement of the equivalent dose in quartz using a regenerative-dose single-aliquot protocol. *Radiat. Meas.* **29,** 503–515 (1998).

3. Murray, A. S. & Wintle, A. G. Luminescence dating of quartz using an improved single-aliquot regenerative-dose protocol. *Radiat. Meas.* **32,** 57–73 (2000).

4. Craig, H. & Gordon, L. I. Deuterium and oxygen 18 variations in the ocean and the marine atmosphere. (1965).

5. Dixit, Y., Hodell, D. A., Sinha, R. & Petrie, C. A. Oxygen isotope analysis of multiple, single ostracod valves as a proxy for combined variability in seasonal temperature and lake water oxygen isotopes. *J. Paleolimnol.* **53,** 35–45 (2015).

6. Gonfiantini, R. Environmental isotopes in lake studies. *Handb. Environ. Isot. Geochemistry; Terr. Environ.* 113–168 (1986).

7. Majoube, M. Fractionnement en oxygene 18 et en deuterium entre l’eau et sa vapeur. *J. Chim. Phys.* **68,** 1423–1436 (1971).

8. Saini, H. S., Tandon, S. K., Mujtaba, S. A. I. & Pant, N. C. Lake deposits of the northeastern margin of Thar Desert: Holocene(?) Palaeoclimatic implications. *Curr. Sci.* **88,** 1994–2000 (2005).

9. Marquart, M., Deisenhofer, J., Huber, R. & Palm, W. Crystallographic refinement and atomic models of the intact immunoglobulin molecule Kol and its antigen-binding fragment at 3.0 Å and 1.9 Å resolution. *J. Mol. Biol.* **141,** 369–391 (1980).

10. Blaauw, M. & Christen, J. A. Flexible paleoclimate age-depth models using an autoregressive gamma process. *Bayesian Anal.* **6,** 457–474 (2011).

11. Reimer, P. J. *et al.* IntCal13 and Marine13 radiocarbon age calibration curves 0–50,000 years cal BP. *Radiocarbon* **55,** 1869–1887 (2013).

12. Dixit, Y., Hodell, D. A. & Petrie, C. A. Abrupt weakening of the summer monsoon in northwest India ~4100 yr ago. *Geology* **42,** 339–342 (2014).

13. Deotare, B. C. *et al.* Palaeoenvironmental history of Bap-Malar and Kanod playas of western Rajasthan, Thar desert. *J. Earth Syst. Sci.* **113,** 403–425 (2004).

14. Enzel, Y. *et al.* High-resolution Holocene environmental changes in the Thar Desert, northwestern India. *Science (80-. ).* **284,** 125–128 (1999).

15. Singh, G., Wasson, R. & Agrawal, D. Vegetational and seasonal climatic changes since the last full glacial in the Thar Desert, northwestern India. *Rev. Palaeobot. Palynol.* **64,** 351–358 (1990).

16. Sinha, R. *et al.* Late Quaternary palaeoclimatic reconstruction from the lacustrine sediments of the Sambhar playa core, Thar Desert margin, India. *Palaeogeogr. Palaeoclimatol. Palaeoecol.* **233,** 252–270 (2006).

17. Gázquez, F., Evans, N. P. & Hodell, D. A. Precise and accurate isotope fractionation factors (α17O, α18O and αD) for water and CaSO4·2H2O (gypsum). *Geochim. Cosmochim. Acta* **198,** 259–270 (2017).

1. * Corresponding author

   E-mail address: yamadixit@gmail.com [↑](#footnote-ref-1)
